# Effectiveness of Phaco-chop, Stop&Chop, and the nitinol filament nuclear disassembly techniques for dense cataract removal

**DOI:** 10.1186/s12886-026-04742-8

**Published:** 2026-03-19

**Authors:** Diego Zalapa, Juan Pablo Herrera-Espinosa, Emilia Ramos-Barrera, Carolina Cantú-Rosales, Sabrina Munita, Roberto González-Salinas

**Affiliations:** Asociación para Evitar la Ceguera en México I.A.P., Anterior Segment Surgery Department,Vicente García Torres 46, Barrio San Lucas, Coyoacán, Mexico City, CP 04030 Mexico

**Keywords:** Nitinol filament nuclear disassembly, Phacoemulsification, Cumulative dissipated energy (CDE), Dense cataracts

## Abstract

**Purpose:**

To compare the effectiveness and outcomes of three surgical techniques for dense cataract removal: Nitinol filament mechanical phaco-fragmentation (NFMP), Phaco-chop, and Stop&Chop by evaluating their impact on ultrasound energy consumption and key perioperative parameters.

**Methods:**

This is a single-center non-randomized retrospective study of patients with dense cataracts who underwent phacoemulsification using NFMP, Phaco-chop, or Stop&Chop techniques. The primary outcome was cumulative dissipated energy (CDE). Secondary outcomes included aspiration time, irrigation fluid usage, phacoemulsification time, endothelial cell loss, and postoperative inflammation. Patients with endothelial cell counts < 2,000 cells/mm^2^, pseudoexfoliation syndrome, uncontrolled glaucoma, zonular weakness, or prior ocular surgeries were excluded.

**Results:**

The study included 105 eyes, distributed across three groups. Mean CDE was lowest in the NFMP group (7.90±3.16), followed by Phacochop (9.25±3.13) and Stop&Chop (10.79±3.94); a statistically significant reduction was found between NFMP and Stop&Chop (*p* < 0.05). Mean phacoemulsification time with NFMP was significantly shorter (40.31±31.71 s) than with Phaco-chop (79.18±34.20 s) or Stop&Chop (80.84±36.76 s) (*p* < 0.01).

**Conclusions:**

Mechanical phaco-fragmentation with NFMP achieved significantly lower energy consumption compared to Stop&Chop, with safety and fluidics comparable to those of conventional techniques. These findings align with the existing literature, which shows the benefits of NFMP in challenging cataract cases; however, the magnitude and significance of the effects may vary depending on patient selection and cataract complexity.

**Clinical trial number:**

Not applicable.

**Supplementary information:**

The online version contains supplementary material available at 10.1186/s12886-026-04742-8.

## Key messages

What is known: The mechanical phaco-fragmentation with nitinol filament (NFMP) has consistently shown reduced cumulative dissipated energy (CDE) and fewer intraoperative adverse events, while achieving comparable visual and refractive outcomes compared to standard phacoemulsification techniques.

New information in the paper: Our study is among the first to compare NFMP with two different techniques as a control group (Stop&Chop and Phaco-Chop). NFMP showed increased CDE reduction versus both techniques; nonetheless, statistical significance was achieved only against Stop&Chop, but not with Phaco-chop, contrasting with prior studies that found significant reductions across all comparisons.

New information in the paper: NFMP led to a significant reduction in mean phacoemulsification time versus both conventional techniques, highlighting improved surgical efficiency and a potential advantage for complex cataract cases.

## Introduction

Phacoemulsification is the gold standard for cataract removal in the developed world. Technological innovation continues to enhance efficiency and improve patient safety. The development of advanced phacoemulsification systems, parameter configurations, tips, and handpieces has provided options for addressing challenging cataract cases, including those with dense cataracts. Dense, brunescent lenses indicate advanced cataracts that have enlarged and hardened over time. Surgical management of these mature cataracts is often increasingly challenging due to factors such as weakened zonules, reduced corneal endothelial cell counts, and shallow anterior chambers, which imply a higher risk of complications during the procedure [1]. As highlighted by Crispim et al., success depends on meticulous preoperative assessment, the use of advanced viscoelastics, optimized phaco techniques (especially chopping methods), and readiness to convert to extracapsular extraction if needed [2].

Endothelial cell loss was initially increased in eyes with a higher nuclear density due to elevated amounts of cumulative dissipated energy (CDE), longer aspiration times, and higher volumes of balanced salt solution (BSS) used during the procedure. The ultrasound energy used during the surgical procedure, as well as the ocular tissue induced damage, can be decreased by employing the burst and pulse modes compared to the continuous mode for dense cataracts [3].

Current phacoemulsification techniques can also help reduce their energy use. Several reports in the literature suggest that the horizontal Phaco-chop technique requires lower ultrasound energy for nuclear management than the Stop&Chop technique in hard or high-density cataracts. However, it has also been reported that the resulting endothelial cell loss is similar with both methods in small-incision cataract surgery [4]. Several other techniques have been introduced in cataract surgery to increase the effectiveness of phacoemulsification of hard cataracts. However, there is no consensus about their efficiency [4].

The microinterventional lens fragmentation method utilizes a disposable, pen-shaped, single-use handheld device called nitinol filament mechanical phacofragmentation (NFMP) (miLOOP® Carl Zeiss Meditec AG). This device employs a microfilament loop composed of a nickel–titanium alloy (nitinol), which expands under the surgeon’s control to capture the lens. Once in place, the loop contracts, enabling complete full-thickness fragmentation of the lens [5].

This study aims to compare the effectiveness and outcomes of three surgical techniques for dense cataracts: 1. NFMP, 2. the Phaco-chop technique, and 3. the Stop&chop technique, based on their performance, as measured by the ultrasound energy used. Few comparative studies have evaluated NFMP simultaneously against both conventional chopping techniques within a uniform surgical context. This study provides novel insight by isolating technique-specific efficiency differences among dense cataracts.

## Methods

### Design and setting

This retrospective non-randomized single-center study included consecutive eyes of patients with cataracts classified as NO4 NC4 or higher, using the Lens Opacities Classification System (LOCS) III [6]^,^ who underwent phacoemulsification with intraocular lens (IOL) implantation at the Anterior Segment Surgery Department at the *Asociación Para Evitar la Ceguera* (APEC), Mexico City, Mexico, between January 2021 and November 2024. All surgeries were performed on the same phacoemulsification platform, the Centurion Vision System (Alcon, Fort Worth, TX, USA). This study was approved by the Ethics Committee and Internal Review Board of the Asociación para Evitar la Ceguera, with grant number MFS-SA-23, and conducted in accordance with the Declaration of Helsinki and Good Clinical Practices Guidelines. As this was a retrospective study of previously collected, anonymized clinical data, the requirement for individual informed consent to participate was waived by the Ethics Committee and Internal Review Board of the Asociación para Evitar la Ceguera. Clinical trial number: not applicable.

### Patients

Patients eligible for this study included men and women aged 40 years or older with hard cataracts classified as ≥NO4 on the LOCS III Scale who underwent cataract surgery using one of the following surgical techniques: NFMP, Phaco-chop, or Stop&Chop. A single experienced surgeon performed all surgeries. The three nuclear disassembly techniques are routinely used in our Anterior Segment Surgery Department for dense cataracts, and in this retrospective series the technique for each case was selected at the discretion of the single-operating surgeon, according to individual preference and experience rather than predefined allocation criteria. The study included healthy patients who attended for a comprehensive evaluation. A detailed ophthalmological assessment was conducted, during which biometry and clinical data were obtained, including visual acuity, axial length, keratometry, white-to-white (WTW) measurement, intraocular pressure (IOP), biomicroscopy, and fundus evaluation. Key inclusion criteria included adult patients with refractive errors ranging from +6.00 to −10.00 D, and regular astigmatism below 5.00 D, and a vision logarithm of the minimal angle of resolution (logMAR) of <1.3. Patients were excluded if they had an endothelial cell count below 2000 cells/mm^2^, pseudoexfoliation syndrome, uncontrolled glaucoma, zonular weakness, a history of ocular trauma, previous eye surgeries, or incomplete medical records. These criteria were set to minimize variables that could affect surgical outcomes and ensure the safety and welfare of the participants.

### Main outcome measure

The primary outcome variable for this study was the energy used during the phacoemulsification procedure, quantified by the CDE count, which represents the total ultrasonic energy delivered to the eye, closely associated with the potential risk of intraoperative damage to ocular structures, particularly the corneal endothelium. Lower CDE values indicate increased efficiency in lens fragmentation and emulsification, which is particularly crucial in dense cataract cases that often require greater energy expenditure.

Secondary variables evaluated included aspiration time (TA), estimated fluid usage, ultrasound (US) time, and loss of corneal endothelial cells. Aspiration time refers to the duration during which lens material is actively removed from the eye. At the same time, estimated fluid usage is an indicator of the volume of balanced salt solution (BSS) employed during the surgery. Both parameters are essential considerations as prolonged aspiration and fluid use can lead to increased endothelial stress and postoperative inflammation. Endothelial cell loss was assessed through pre- and postoperative specular microscopy. The collected data was captured on an Excel spreadsheet (Microsoft ® Corporation, Washington, USA) for further analysis.

### Statistical analysis

Continuous and categorical variables are shown as means ± standard deviation (SD) and percentages, respectively. The Ordinary one-way ANOVA test was used for group comparisons, while the Kruskal-Wallis test was used when the data distribution was not normal. Additionally, Tukey or Dunn´s post hoc multiple-comparison analyses were performed to identify specific intergroup differences based on data distribution. Variable distributions were obtained using the Shapiro-Wilk test. The statistical analysis was performed using SPSS software version 20, and figures were created using GraphPad PRISM version 8.

### Sample-size calculation

The sample size for this study was determined using the two-means comparison formula. The sample-size calculation utilized the standard deviation of the mean CDE values reported previously in our institution for dense cataracts [7]. Our previous internal data for dense cataracts showed a mean CDE of 9.6 ± 3.1, which served as the basis for the present sample size estimation. The significance level (α) employed was 0.05 and a statistical power (1-β) of 80%, with corresponding Z-values of 1.96 and 0.84, respectively. The required sample size per group was calculated as follows:

*N* = 2 × s^2^ × (Zα + Zβ)^2^/Δ^2^

*N* = 2 × (2.90)^2^ × (1.96 + 0.84)^2^/(2.25)^2^ ≈ 34.89 subjects per group

A total of 105 participants across the three groups was deemed sufficient for the study.

## Results

A total of 105 eyes of 105 patients were included, of which 62 (59.05%) were female patients; demographic data is shown on Table 1. The total of patients was separated into three groups: the first group underwent cataract removal by phacoemulsification using the mechanical phacofragmentation with a nitinol filament technique for nuclear disassembly (Group 1), the second employed the horizontal Phaco-chop technique (Group 2), and the third used the Stop&Chop technique (Group 3), with the same surgical parameters: longitudinal power 40%, torsional 60%, vacuum 380 mmHg; for all patients.

**Table 1:** Patient demographics and baseline ocular characteristics across surgical groups

Parameter	Group 1	Group 2	Group 3	P value
**N (%)** n = 105	35 (33.3%)	35 (33.3%)	35 (33.3%)	–
**Age (years)** Mean ± SD 95% CI	71.67 ± 8.85 67.93–78–2	77.30 ± 7.91 62.1–69.79	70.08 ± 9.59 64.46 - 75.54	0.123
**AL (mm)** Mean ± SD 95% CI	23.1 ± 0.93 23.02–23.57	23.45 ± 0.91 23.21–24.0	23.33 ± 0.81 23.42–23.89	0.745
**ACD (mm)** Mean ± SD 95% CI	3.23 ± 0.71 2.69–3.54	3.08 ± 0.89 2.59–3.66	3.34 ± 0.76 2.71–3.77	0.875
**LT (mm)** Median ± SD 95% CI	4.41 ± 0.62 3.82–4.41	4.29 ± 0.11 3.98–4.61	4.31 ± 0.41 4.11–5.21	0.602
**Km** Mean ± SD 95% CI	44.14 ± 2.01 43.42–45.01	43.99 ± 1.97 42.99–44.89	42.20 ± 1.76 43.19–44.96	0.640

*One-way ANOVA test

SD = Standard deviation

**Fig. 1:**
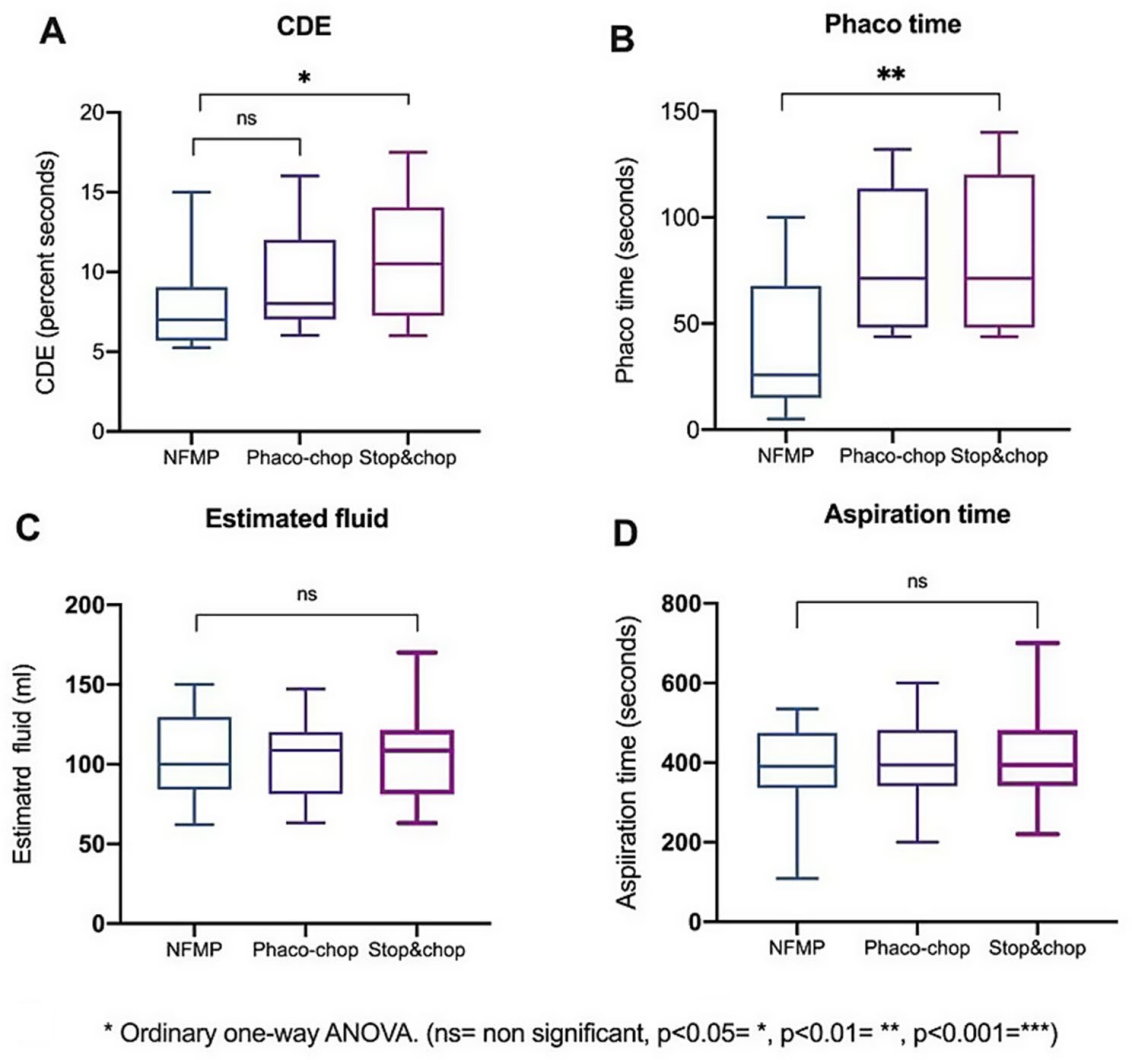
Comparison of surgical parameters across three phacoemulsification techniques. Box plots display median, interquartile range, and full data range for (**A**) CDE, (**B**) phacoemulsification time, (**C**) estimated fluid usage, and (**D**) aspiration time. Ordinary one-way ANOVA: *p < 0.05, **p < 0.01; ns = not significant. CDE = cumulative dissipated energy; NFMP nitinol filament microinterventional prefragmentation

The CDE used during surgery varied among the three groups, as demonstrated in Fig. 1. Group 1 had a mean CDE of 7.90 ± 3.16 with a range of 9.74, Group 2 showed a mean CDE of 9.25 ± 3.13 with a range of 10.0, and Group 3 exhibited the highest mean CDE at 10.79 ± 3.94 with a range of 11.50. Notably, the comparison between the NFMP and Stop&Chop groups reached statistical significance (*p* < 0.05). The difference between the NFMP and Phaco-chop groups was not statistically significant.

The phacoemulsification time differed markedly among the surgical techniques. The mean phacoemulsification time was lowest in Group 1 at 40.31 ± 31.71 seconds compared to 79.18 ± 34.20 seconds in Group 2 and 80.84 ± 36.76 seconds in Group 3. The median phacoemulsification times similarly reflected this pattern, with NFMP at 25.85 seconds, and both Phaco-chop and Stop&Chop at 71.30 seconds. Statistical analysis confirmed a significant reduction in phacoemulsification time for NFMP compared to both conventional techniques (*p* < 0.01).

The estimated volume of irrigation fluid used during surgery was comparable across the three groups. Statistical analysis confirmed that there were no significant differences among groups (*p* > 0.05). Similarly, aspiration time did not differ significantly among the surgical techniques. Mean aspiration times were around 400 seconds for all three groups, with ranges spanning approximately 100 to 550 seconds for NFMP. No statistically significant differences were found in aspiration time across groups.

Although the NFMP technique demonstrated lower mean CDE and shorter phacoemulsification times, statistically significant differences were found only against the Stop‑and‑Chop group, likely reflecting intra‑group variability in surgical time and energy use.

**Fig. 2:**
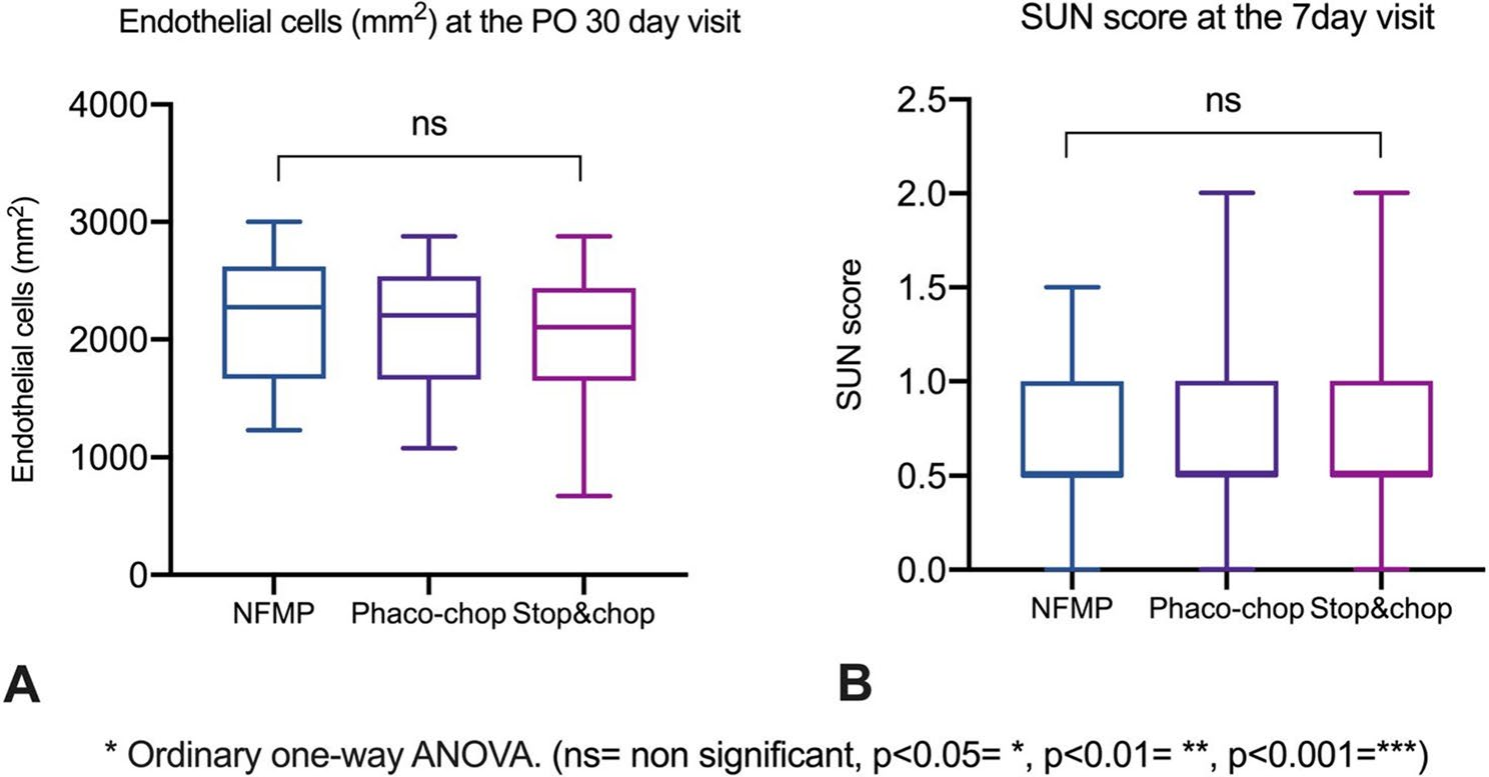
Safety outcomes comparing NFMP, Phacochop, and Stop & chop techniques. Box plots display (**A**) Corneal endothelial cell density at postoperative day 30, and (**B**) Anterior chamber inflammation assessed by Standardization of uveitis nomenclature (SUN) score at postoperative day 7. Statistical analysis performed using one-way ANOVA. NFMP = nitinol filament microinterventional prefragmentation

At 30 days postoperatively, the corneal endothelial cell count (CCE) was comparable across the three surgical groups. Mean CCE values were similar, with overlapping standard deviations and no apparent outliers favoring any group. We also assessed the degree of postoperative inflammation in the anterior chamber (CA) at day 7 for each group. The mean inflammation was consistent across the three surgical techniques, with central values around grade 2 of cellularity according to the Standardization of Uveitis Nomenclature (SUN) classification for cells in the anterior chamber [8]. Statistical analysis confirmed no significant differences in anterior chamber inflammation between the surgical methods, as depicted in Fig. 2.

**Fig. 3:**
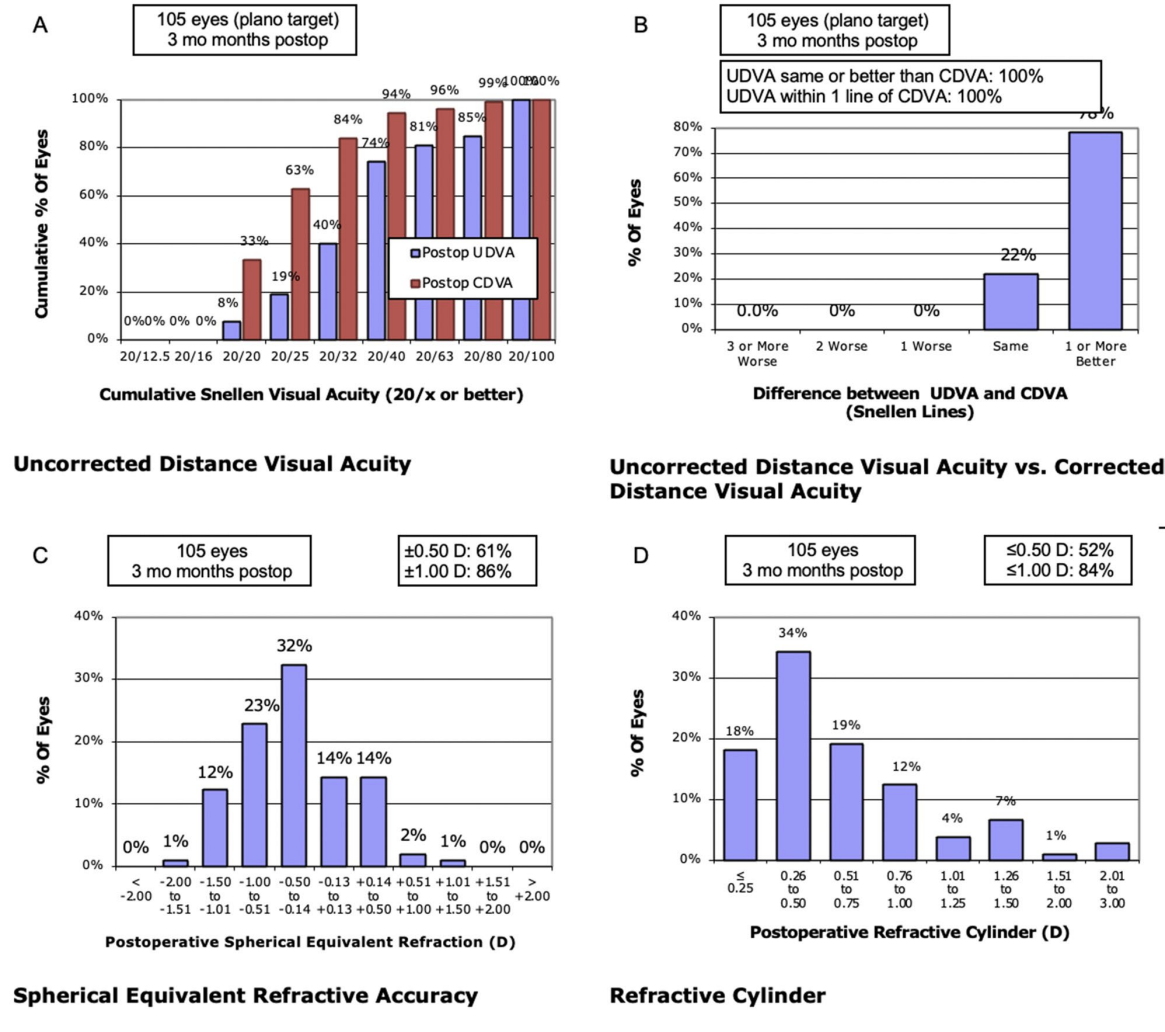
Standard four graphs for cataract refractive outcomes at 3 months postoperatively standard four graphs for cataract refractive outcomes for all 105 eyes assessed at 3 months postoperatively. Panel **A** shows the cumulative distribution of uncorrected distance visual acuity (UDVA) and corrected distance visual acuity (CDVA) in Snellen lines. Panel **B** depicts the difference between UDVA and CDVA in Snellen lines. Panel **C** illustrates the distribution of postoperative spherical equivalent refraction and the proportion of eyes within ±0.50 D and ±1.00 D of the intended target. Panel **D** shows the distribution of postoperative refractive cylinder and the proportion of eyes with residual astigmatism

In the analysis of refractive outcomes, we constructed the standard four graphs for cataract refractive outcomes to comprehensively evaluate the refractive results (Fig. 3) .

## Discussion

The development and introduction of the NFMP device have advanced mechanical endocapsular lens fragmentation in cataract surgery, enabling full-thickness segmentation of the nucleus without the need for phacoemulsification energy. This novel approach utilizes a superelastic nitinol loop, enabling safe and efficient division of the lens independent of cataract grade, reducing stress on the capsular bag and zonules compared to ultrasound chopping methods [9].

In our study, we found that the CDE, the primary marker of ultrasound energy delivered during cataract surgery, was numerically lowest in Group 1. However, statistical significance was only observed when comparing the NFMP and the Stop&Chop groups, while the difference between the NFMP and Phaco-Chop groups did not reach significance. This suggests a clear trend towards lower energy utilization when using the NFMP technique, but also highlights potential variability depending on surgical approach and the patient cohort. The existing literature consistently reports statistically significant CDE reductions when using the NFMP technique. For example, Hu et al. [1] demonstrated a 21% lower mean CDE with NFMP compared to conventional phacoemulsification in dense cataracts (9.6±5.2 vs 11.6 ± 6.4, *p* < 0.0001). Similarly, Ianchulev et al. reported mean CDE values of 21.4 ± 13.1 with NFMP and 32.8 ± 24.9 in controls (*p* = 0.004) among patients with high nuclear density [10]. Wiley et al. also found significant reductions, with a 16–23% decrease in phacoemulsification time or energy when applying the nitinol filament device in general and dense cataract cohorts [5]. The Nitinol filament device mechanically fragments the lens nucleus before ultrasound application, allowing for more efficient phacoemulsification and minimizing the ultrasound energy applied to ocular tissues. This is especially critical in dense and more advanced cataracts, where traditional techniques require substantially more energy, increasing the risk of corneal endothelial damage and postoperative complications. Our findings, while not statistically significant across all group comparisons, align directionally with these studies and underscore the clinically significant upside of incorporating the NFMP technique to optimize both intraoperative efficiency and postoperative safety. A comprehensive CDE comparison between nuclear disassembly techniques reported in literature is summarized in Table 2.

**Table 2:** Comparison of cumulative dissipated energy and intraoperative parameters among techniques and prior studies

Study	Design & Population	Phacoemulsification system	NFMP Group CDE (mean±SD)	Control Group CDE (mean±SD)	% Reduction	p-value
Zalapa et al. (Our study)	Retrospective, single-surgeon, single-center, dense cataracts	Centurion Vision System (Alcon)	7.90 ± 3.16	9.25 Phaco-chop, 10.79 Stops&Chop	15–25	NFMP vs. Stop&Chop <0.05
Hu et al. 2022 [1]	Retrospective, dense cataracts	Centurion Vision System (Alcon).	9.6 ± 5.2	11.6 ± 6.4	21	<0.0001
Ianchulev et al. 2019 [10]	Prospective multi-surgeon randomized controlled trial in dense cataracts	Centurion Vision System (Alcon)	21.4 ± 13.1	32.8 ± 24.9	53	0.004
Wiley et al. 2021 [5]	Single-center, single-surgeon, all cataracts, and dense cataracts	Whitestar Signature Pro (Johnson & Johnson Vision)	9.5 ± 8.5 (all) 11.8 ± 9.5 (dense)**	11.3 ± 10.5 (all) 15.3 ± 12.6 (dense)*	16 (all) 23 (dense)	<0.05

**Measured energy usage as the absolute phacoemulsification time, not CDE. CDE = cumulative dissipated energy; NFMP = nitinol filament mechanical fragmentation

Mean fluid usage was lowest in Group 1, nonetheless remained comparable across all three surgical techniques, with no statistically significant differences between groups. These findings indicate that, within our sample, the use of the NFMP device did not substantially alter surgical fluidics compared to conventional approaches. In contrast, published literature has reported substantial improvements in fluidics when using NFMP, especially in more challenging cases. For instance, Ianchulev et al. observed that eyes treated with NFMP required significantly less irrigation fluid (87.3±47.0 mL) compared to controls (111.2±55.7 mL) [10]. One possible explanation for the lack of significant differences in our cohort may be the exclusion of patients with endothelial cell counts below 2000 cells/mm^2^, pseudoexfoliation syndrome, uncontrolled glaucoma, zonular weakness, and those with a history of ocular trauma or previous eye surgeries. By selecting a lower-risk population, our study may have reduced the variability and potential for complications or inefficiencies that tend to amplify the benefits of the NFMP technique in fluidics, as seen in other studies that included higher-risk or more complex eyes.

In our study, the NFMP-assisted cataract surgery resulted in the lowest mean phacoemulsification time (40.31±31.71 seconds) compared to Phaco-Chop (79.18±34.20 seconds) and Stop&Chop (80.84±36.76 seconds), with a statistically significant reduction observed for the NFMP group versus both conventional techniques (*p* < 0.01). These findings align with the results from Wiley et al., who reported that using NFMP led to a 42% reduction in total phacoemulsification time, with efficiency benefits increasing to 49 and 57% in dense cataracts, respectively (all *p* < 0.02) [5].

We found that the magnitude of the corneal endothelial cell loss was similar across all surgical groups one month following the procedure, which is consistent with prior research. In this regard, Hu et al. reported similar rates of significant corneal edema (Grade 3+) in both control and NFMP device groups (5.5% vs 5.0%, respectively) [1]. Similarly, Ianchulev et al. found that endothelial cell loss averaged less than 10% at one month with no group difference [10]. While Jung Hyun Park et al. did not evaluate NFMP, their comparative analysis of phaco-chop and stop-and-chop techniques according to nuclear density revealed that, for dense cataracts, both techniques resulted in similar endothelial cell loss and postoperative corneal thickness [11]. This further supports the idea that multiple modern phaco-techniques can achieve comparable safety outcomes with respect to endothelial preservation, even as differences emerge in energy efficiency for dense nuclei. The apparent lack of correlation between lower CDE and endothelial cell loss in our series may be explained by several factors. Endothelial damage after phacoemulsification is multifactorial and depends not only on ultrasound energy but also on fluidics, anterior chamber depth, nuclear density, and pre-existing endothelial reserve [12]. Also, endothelial cell density was assessed only at the one-month follow-up visit; thus, longer‑term studies are needed to assess endothelial changes beyond this time frame.

Recent comparative studies further reinforce the energy and safety benefits of mechanical chop techniques in dense cataract surgery. Singh et al. demonstrated that, among patients with grade 3–4 nuclear cataracts, direct chop techniques resulted in significantly less endothelial cell loss compared to Stop&chop, highlighting the importance of minimizing phaco energy and intraocular trauma in complex cases [13]. Similarly, Fernández-Muñoz et al. found that Phaco-chop yielded the lowest CDE values among three fragmentation techniques (Phaco-chop, divide-and-conquer, and ultrachopper) for dense cataract removal and achieved comparable postoperative endothelial cell densities across groups [14]. These findings collectively support the trend observed in our study, emphasizing that energy-efficient mechanical fragmentation, via NFMP or conventional Phaco-chop approaches, can optimize both intraoperative safety and long-term corneal health when managing dense cataracts.

Furthermore, in our study, we observed no surgical or postoperative complications across all groups, including those in which the NFMP device was used. This finding stands out especially when considering existing literature. Hu et al. reported a significantly lower intraoperative complication rate with the NFMP technique (2.2% vs. 6.3% for controls, *p* = 0.001), but still encountered some adverse events in both groups [1]. Similarly, Ianchulev et al. found comparable complication rates between NFMP and standard phacoemulsification (7.5% vs. 10.4% capsular tears, respectively) in a high-risk, advanced cataract population. [10] In contrast, Wiley and his research group reported no increase in adverse events when employing the NFMP-assisted surgery, although they did not have a zero-complication cohort [5]. The absence of complications in our series may be attributed to the fact that all procedures were performed by a single, experienced surgeon. Also, the patients had dense cataracts, lacking complicated features such as zonular damage, pseudoexfoliation, and glaucoma, which, in turn, likely contributed to optimized surgical outcomes.

Refractive results demonstrated high levels of uncorrected and corrected distance visual acuity, with most eyes achieving postoperative spherical equivalent within ±0.50 D and low residual refractive cylinder, underscoring the overall refractive effectiveness of surgery in dense cataracts. These findings are in line with previous reports suggesting that optimized nuclear disassembly and energy‑modulation strategies may contribute to improved refractive predictability. For example, Roper et al. reported that eyes undergoing NFMP‑assisted surgery achieved a median absolute refractive error of 0.10 D compared with 0.19 D in controls (*p* = 0.002), with a higher proportion of cases reaching the intended target refraction, supporting the concept that refinements in technique can translate into better refractive outcomes [15]. Although some patients achieved good refractive accuracy and improved best‑corrected visual acuity, their final vision remained partially limited by coexisting macular pathology such as diabetic macular edema and age‑related macular degeneration, which may have attenuated the functional gains attributable solely to the cataract procedure.

Additionally, Vivekanandan et al. recently described a novel technique combining NFMP-assisted nucleotomy with IOL scaffold implantation for hypermature cataracts with liquefied cortex. By implanting an IOL beneath the undivided nucleus after decompressing the bag, the scaffold technique stabilizes the nucleus and protects the posterior capsule during NFMP-assisted nucleotomy. In their series of 32 cases, this approach successfully minimized capsular and zonular trauma, allowing safe and efficient division of brunescent nuclei even in challenging eyes at high risk for complications [16].

Our study had several limitations that should be considered when interpreting the results. First, its retrospective design may introduce inherent biases related to the selection and recording of data. Second, although the sample size was determined through a pre-study calculation based on the primary outcome variable and was fully achieved, the overall number of participants remains relatively limited, which may affect the generalizability of the findings. Third, there was a 5% loss of data due to incomplete patient records. To address this, a systematic approach was employed to manage missing data by using the average of available observations, which helps maintain the validity of the dataset; however, it may still introduce some degree of estimation bias. Overall surgical time was not systematically recorded for all procedures, preventing a detailed comparison of total operative duration between techniques. Finally, the absence of longer‑term endothelial evaluations limits our ability to determine whether the modest cell loss observed at the one-month postoperative visit remains stable, improves, or progresses over time. Without follow‑up measurements at later intervals (such as 3 or 6 months), any conclusions about the sustained safety of the techniques with respect to corneal endothelial preservation must be interpreted with caution. Despite these constraints, the study provides valuable insights into surgical parameters associated with the NFMP technique.

Our study demonstrates that NFMP-assisted phacoemulsification offers a significant reduction in CDE compared to the Stop&Chop technique, while maintaining surgical efficiency, safety, and corneal endothelial preservation comparable to both Phaco-chop and Stop&Chop. By excluding patients with added ocular risk factors, we were able to assess the effect of surgical technique individually. Our results expand prior evidence by demonstrating that NFMP delivers significant time and energy savings over Stop&Chop while maintaining safety comparable to Phaco‑chop in normal dense cataracts. Nonetheless, cases including pseudoexfoliation, uncontrolled glaucoma, previous trauma, and prior surgery should be assessed in further studies. Therefore, further randomized prospective studies are warranted to confirm these findings.

## Electronic supplementary material

Below is the link to the electronic supplementary material.

Supplementary Material 1

## Data Availability

The datasets generated and analyzed during the current study are not publicly available due to restrictions related to institutional data sharing policies but are available from the corresponding author on request.

## Declarations

### AI Use

During the preparation of this work, the authors used Grammarly to improve the grammar and clarity of the manuscript text. After using this tool, the authors reviewed and edited the content as needed and take full responsibility for the content of the publication.
